# Does influenza A virus infection affect movement behaviour during stopover in its wild reservoir host?

**DOI:** 10.1098/rsos.150633

**Published:** 2016-02-10

**Authors:** Daniel Bengtsson, Kamran Safi, Alexis Avril, Wolfgang Fiedler, Martin Wikelski, Gunnar Gunnarsson, Johan Elmberg, Conny Tolf, Björn Olsen, Jonas Waldenström

**Affiliations:** 1 Centre for Ecology and Evolution in Microbial Model Systems (EEMiS), Linnaeus University, Kalmar 391 82, Sweden; 2 Deparment of Migration and Immuno-Ecology, Max Planck Institute for Ornithology, Am Obstberg 1, Radolfzell 78315, Germany; 3 Department of Biology, University of Konstanz, Konstanz 78457, Germany; 4 Division of Natural Sciences, Kristianstad University, Kristianstad 291 88, Sweden; 5 Section of Infectious Diseases, Department of Medical Sciences, Uppsala University, Uppsala 751 85, Sweden; 6 Zoonosis Science Centre IMBIM, Uppsala University, Uppsala 751 23, Sweden

**Keywords:** avian influenza A virus, effect of infection, mallard, movement, stopover, transmission

## Abstract

The last decade has seen a surge in research on avian influenza A viruses (IAVs), in part fuelled by the emergence, spread and potential zoonotic importance of highly pathogenic virus subtypes. The mallard (*Anas platyrhynchos*) is the most numerous and widespread dabbling duck in the world, and one of the most important natural hosts for studying IAV transmission dynamics. In order to predict the likelihood of IAV transmission between individual ducks and to other hosts, as well as between geographical regions, it is important to understand how IAV infection affects the host. In this study, we analysed the movements of 40 mallards equipped with GPS transmitters and three-dimensional accelerometers, of which 20 were naturally infected with low pathogenic avian influenza virus (LPAIV), at a major stopover site in the Northwest European flyway. Movements differed substantially between day and night, as well as between mallards returning to the capture site and those feeding in natural habitats. However, movement patterns did not differ between LPAIV infected and uninfected birds. Hence, LPAIV infection probably does not affect mallard movements during stopover, with high possibility of virus spread along the migration route as a consequence.

## 1. Introduction

The dynamics between a pathogen and its host are key to the evolution and spread of the pathogen. In some cases, the cost for the infected host may be negligible [1–3], but typically it is not. In fact, even small reductions in host condition can have severe consequences with respect to lowered fitness. This is particularly the case when survival or breeding success depends on peak performance, which is why natural selection in hosts promotes a response that reduces the cost of infection [4]. Selection on pathogens, on the other hand, promotes their transmission [5]. High virulence and explosive spread may thus be a successful strategy, but if the effect on the host is severe (especially, if lethal) the chances of effective long-term transmission decrease substantially. Instead, causing mild symptoms may result in lower pathogen reproduction within an individual host. In return, this could maximize spread and thus be optimal from a pathogen perspective [6]. Although the generality of such a model has been questioned [7], a strong trade-off effect is generally assumed between virulence and transmission [8].

Pathogen transmission may be greatly enhanced if the host undertakes long movements. Long-distance migration as part of the seasonal or annual programme is a striking example, and one that often involves extraordinary physical effort. This, in turn, may necessitate temporary re-allocation of energy resources during the migration period [9]. Therefore, migrating animals may face a particularly challenging situation, in which migration effort can reduce immune system functioning and lead to increased susceptibility to pathogens [10]. Consequently, animal migration may play a key role in pathogen transmission, as migratory hosts can spread pathogens into new areas (and onto new hosts). From the host perspective, selection will promote less virulent pathogens, as hosts carrying more virulent strains are less likely to perform migration successfully [10].

Wild birds, particularly waterbirds such as ducks, waders and gulls (orders Anseriformes and Charadriiformes), are the main hosts of influenza A virus (IAV) in nature. Most waterbird taxa are migratory and highly social during the non-breeding season, both of which are features that promote pathogen spread. In North America and Eurasia, dabbling ducks (genus *Anas*) stand out as particularly important reservoir hosts, associated with high prevalence rates and large virus subtype diversity [11,12]. The most numerous and best studied species is the mallard (*Anas platyrhynchos*), in which a large proportion of all IAV subtype combinations known to exist in birds have been recorded [13–15]. While studies addressing the effects of IAV infection in wild mallards are still rare, they are urgently required in order to understand the evolution and epidemiology of IAV, as well as its ecological consequences for the duck host.

Migration patterns differ among mallard populations, northern birds generally being migratory and southern being mainly sedentary [16]. Thus, the epidemiology of IAV is potentially affected by migration, allowing spread of viruses over considerable distances during annual migrations, e.g. from the breeding grounds in the boreal biome to wintering areas at more southerly latitudes (e.g. temperate regions) [17–21]. Although it has been hypothesized that IAV can be maintained in the ambient environment for several months [22], IAV dynamics in arctic-temperate regions suggest that the virus cannot be maintained year round at the northernmost breeding grounds [23,24]. As a consequence, the prevalence of IAV at higher latitudes usually peaks in autumn instead of summer [25–27], when immunologically naive juveniles are exposed as they gather in flocks at stopover sites [11]. Under such circumstances IAV prevalence may, for short periods, reach 50–60% in autumn [25,28]. As immunity is progressively acquired, prevalence decreases through autumn and winter. Although prevalence is low in spring, often 0–2% [25], it may still be high enough to bring the virus back to the breeding grounds to initiate a new infection cycle.

Infections with the low pathogenic phenotype of IAV (LPAIV) in wild birds are often assumed to be asymptomatic [3,29]. However, LPAIV infections in reared mallards have been shown to cause a small and short-lived increase in body temperature [30]. Moreover, naturally infected wild mallards have been associated with somewhat lower body mass compared with uninfected individuals [31]. A review on IAV virulence in waterbirds [32] hypothesized that LPAIV affects the digestive organs, as occurrence and intensity of infection was negatively correlated with body mass, and that the lower intestine is the primary site for virus replication. Thus, there may be effects on health and possibly also on fitness of LPAIV infected birds. Even small reductions in vigour can have important consequences at the individual level, negatively affecting reproduction as well as survival probability. An LPAIV infection may also influence migration decisions in mallards [33] and manifest itself as reduced mobility during stopovers, to compensate for increased risk of predation and/or increased need for foraging.

Clearly then, there is potential for trade-offs associated with LPAIV infection and migration in mallards. However, to date there is very little research investigating these trade-offs. We, therefore, aimed to compare movements between infected and uninfected mallards in the wild, at a major stopover site in the Northwest European flyway [34]. To achieve this, we equipped autumn-staging mallards of known LPAIV infection status with local read-out GPS transmitters and accelerometers. We hypothesized that LPAIV infection would alter movements and predicted that movement activities should be lower in infected mallards compared with uninfected individuals.

## 2. Material and methods

### 2.1 Study area and trapping

Öland is a Swedish island in the southern Baltic Sea. It is approximately 140 km from north to south and 18 km east to west at the widest. The island is a major stopover site for dabbling ducks and other waterfowl during autumn [35–37]. Trapping was carried out at Ottenby Bird Observatory (56°12′ N, 16°24′ E), where a stationary trap specifically designed to catch wild ducks for ringing and epidemiological studies has been used continuously since 2002. Wild ducks are attracted to the trap by bait grain and lure-ducks (kept in a separate compartment). Once every day, ducks that have entered the trap are herded into a separate section where they are placed in individual cardboard boxes and taken to a field laboratory for further handling. This includes attaching an aluminium ring with a unique identification number around the tarsus, age and sex determination, biometric measurements (e.g. wing length, bill-head, body mass) and influenza screening (see [26] for further details).

### 2.2 Study design and influenza screening

Mallards were caught, sampled and selected for GPS tagging over a period of 3 days (25–27 October 2010) during the peak of autumn migration. All captured mallards were sampled for IAV by fresh faecal samples, if available, or cloacal swabs, the latter method having been reported to yield a lower detectability [38]. The samples were immediately taken to the laboratory, RNA extracted and RRT-PCR amplified for detection of the IAV matrix gene (described in [26]), while the ducks were kept in cardboard boxes at the trap (for 3–5 h). In total, 40 juvenile (first calendar year) mallards were used in the telemetry experiment, of which 24 were males and 16 were females. In terms of LPAIV, 50% of each gender were infected at the beginning of the study.

If mallards were recaptured and sampled as part of the continuous sampling scheme, which continued during the study, the infection status on that day was linked to movements recorded after the recapture. Mallards used in this study changed infection status on average 1.4 times (s.d. = 1.7) and the average daily recapture rate was 0.35 (s.d. = 0.3) for an average monitoring duration of 25 days (s.d. = 13). It should be noted that the studied mallards experienced all the selective pressures and subsequent decision-making processes nature brings, rather than a confined laboratory environment that can possibly mask ecological effects of infection (or result in ecologically irrelevant behavioural artefacts).

### 2.3 GPS and accelerometer devices

Each of the 40 selected mallards was equipped with a Bird 2AA2 (e-obs Digital Telemetry, Grünwald, Germany) device that included a GPS transmitter and three-dimensional accelerometer, and then released at the site of capture. The devices had a maximum measurement of 90×30×16 mm and were equipped with a 70 mm long antenna, angled backwards. Total device weight was 51 g (approx. 5% of mallard body mass). The device was attached with a harness (see [39] for further details) and a 4 mm thick neoprene pad glued to the bottom of the device. The GPS recorded one data point every 15 min, including information about time, location (accurate to within approx. 10 m) and speed over ground. The accelerometer recorded data in three axes every 2 m for approximately 4.2 s with a sampling rate of 18.74 Hz for 24 h. The manufacturer specified battery life of the devices was estimated to be three weeks minimum. In this event, some were still functioning after seven weeks.

### 2.4 Fieldwork

The mallards were tracked for seven weeks using portable download equipment with a receiver (e-obs base station). The distance at which data could be downloaded ranged from 4 km (under ideal conditions) to a few hundred metres, depending on landscape features. For 30 days (25 October–23 November), data were downloaded twice every day (morning and evening) within the nature reserve where Ottenby Bird Observatory (and the duck trap) is situated. To ensure that data were collected from all tagged mallards remaining in the study area, we performed 13 flights (26 October–17 November) along the coast of southern Öland with a light aeroplane, on which a receiver was mounted. On three flying occasions, we also searched the northern part of the island and the mainland coast, without finding any tagged individuals. On days when aeroplane flights were not conducted (mainly due to bad weather), the core area (25–30 km of the east coast and other known staging areas) was covered once a day by car and foot. After 23 November, data collection from the 12 remaining mallards was performed once per day on 25, 27, 28, 30 November and 3, 8, 10 December by car and foot, and once by plane on 8 December. When the study was terminated on 10 December, snow covered the island and only one mallard remained in the study area.

We were able to download data from all but two (i.e. 38) devices, half of which were attached to mallards infected by LPAIV at the start of the study. One device was removed from a recaptured individual (on 9 November), as it had stopped sending signals. The remainder was still attached to mallards as they departed the study area.

### 2.5 Movement metrics and statistical analyses

We used raw data from the accelerometer, i.e. the unfiltered digital readings of the analogue–digital converter of the sensor, for further computation because most loggers were not calibrated and, therefore, linear conversions into the normal dimension of acceleration (m s^−2^) would not have revealed correct values. Movements of the ducks were summarized to overall dynamic body acceleration (ODBA), describing the rate at which animals expend energy [40,41], which can thus be used as a proxy for movements possibly affected by LPAIV infection in mallards. For each burst (approx. 4.2 s every 2 min), signals of the three axes were individually smoothed using running means over 3.8 s. Each unsmoothed data point was then subtracted from the corresponding smoothed data. The sum of all three axes over the total length of the burst then resulted in a single value describing the tri-axial dynamic acceleration experienced, whereby larger values represent more movement of the body. These dynamic acceleration values were then averaged per individual starting from the capture event until the next capture event or a maximum of 7 days. When an individual was recaptured, the time after last release was counted as a new ODBA event. In total, 87 ODBA events were obtained from 37 mallards and the corresponding infection status for each of these periods was determined as described above. To test for differences in the amount of body movements, we compared the ODBA values for each individual in phases when classified as infected with phases when classified as uninfected, using a pairwise *t*-test. Paired values (infected and uninfected status from the same individual) were available for 20 of the 40 mallards in the study.

We computed nine metrics from GPS data (table 1) to describe movement quantitatively and qualitatively for each day/night period during which the mallards were monitored (see day/night behaviour below). We investigated the association between infection (Inf) and movement metrics recorded for each bird using linear mixed models with individuals as random effects to account for repeated measures at the individual level. Some metric distributions were highly skewed towards the right and present an excess of very small values, mainly attributed to periods when the mallards were mostly stationary. Hence, when necessary, the movement metric was log-transformed to satisfy normal residual distribution assumptions before analysis. We also ran the models with different error structures using generalized linear mixed models (GLMM) accounting for positive skewness (inverse Gaussian and gamma distribution) to make sure that our conclusions were not influenced by misspecification of the error distribution. Quantification of movement metrics and all statistical analyses were performed in R v. 3.0 [42] and package lme4 [43]. Error distribution and model assumptions were visually checked using residual plots. In addition, we investigated departures from model assumptions using the appropriate goodness of fit tests for the expected error distribution. Kolmogorov–Smirnov tests were used for checking the normality of errors, whereas Pearson's *χ*^2^ tests were used on the GLMM Pearson's residuals.

**Table 1.** Movement metrics used in the analyses, their significance and abbreviations.

movement metric	explanation	abbreviation
total cumulative distance travelled	the total sum of the lengths of the recorded trajectory measured in metres using great circle distances between all consecutive recorded locations	*D*_tot_
maximum distance	maximum displacement or the maximum distance between any two locations the birds were observed	*D*_max_
average distance	mean of all step lengths measured in metres from consecutive locations	*d*_seg_
mean speed	mean of the step length in metres divided by the time lag in seconds	*v*_seg_
maximum speed	maximum value of distance covered between two locations divided by time	*v*_max_
mean coefficient of the first passage time (FPT)	the FPT measures the time an individual needed to cross a circle of a given radius *r*. It is a cross-scale analysis of the movement pattern, where the slope of the log of the mean of FPT against the log of the radii of the circles should be about 2 for Brownian motion. Lower values indicate facilitated diffusion, or subdiffusive or advective movement, referring to non-random and thus, more or less, directional movement. Higher slopes indicate superdiffusive or impeded diffusion	FPT_coef_
intercept of the mean FPT	the intercept of the correlation of the log of the radii and the FPT is an indicative of the most basal movement characteristic, or the tendency to move irrespective of scaling effects (innate movement)	FPT_int_
dispersion factor of a Brownian bridge based on the trajectory	movement can be described by a conditional Brownian motion where an animal moved between known locations in a conditional Brownian fashion, corresponding to a two-dimensional Gaussian process, with a certain variance or dispersion factor (*h*). The higher the estimated variance, the more erratic the movement was and more deviance around the straight line connection is to be expected	*h*
area of a minimum convex polygon	the area that the animal used according to a minimum area polygon containing all observed locations	MCP

### 2.6 Model structure and explanatory variables

In our model, Inf was a two-level explanatory factor (infected versus uninfected), describing the individual state of infection at the time of release. As LPAIV infections are of short duration (often less than 8 days) [31], we analysed individual movements based on GPS data collected within a week after each release. If a bird was recaptured in the 7 days following its release, the time scale was reset and the 7 days thereafter were considered according to the updated state of infection. To avoid potential handling effects on movement due to capture, we reran the analyses excluding the first (12 h) period following release.

**Figure 1.**
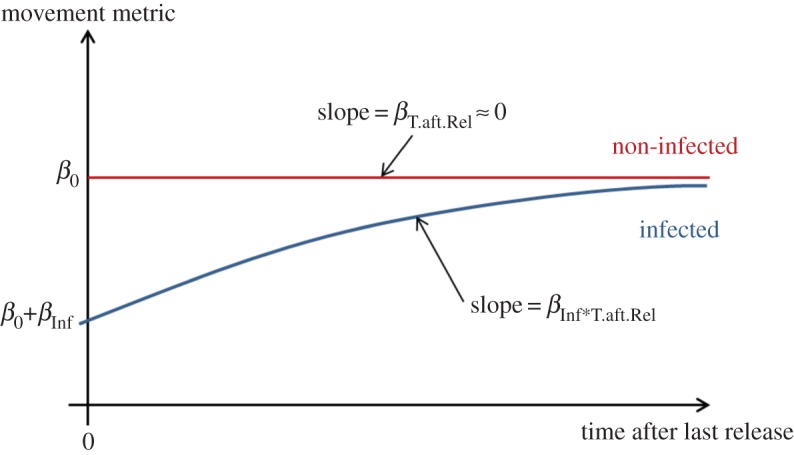
Theoretical predictions of the influence of infection on movement metrics. If infection affects spatial behaviour, infected (blue) and uninfected (red) birds should behave differently at the time of release. We postulate that, at this time, movement metrics for infected birds should be lower than for uninfected birds, which would be revealed as different intercepts of the regression of the movement metrics against time for uninfected (*β*_0_) and infected birds (*β*_0_+*β*_Inf_). As infected birds recover with time, their movement metrics will approach and eventually meet the values for uninfected birds. This happens when the slope of the regression of the movement metrics against time for infected individuals (*β*_T.aft.Rel*inf_) reaches the slope for uninfected birds (*β*_T.aft.Rel_), which is expected to be null.

Time after last release (T.aft.Rel) was included in models as an explanatory continuous covariate. For uninfected individuals, we expected almost no variation in movement metrics with time after release. By contrast, we expected movement metrics for infected birds to change with time following recovery from infection (visualized in figure 1). As a result, we included the interaction Inf*T.aft.Rel to account for these two alternative trends in the movement metric variation.

**Figure 2.**
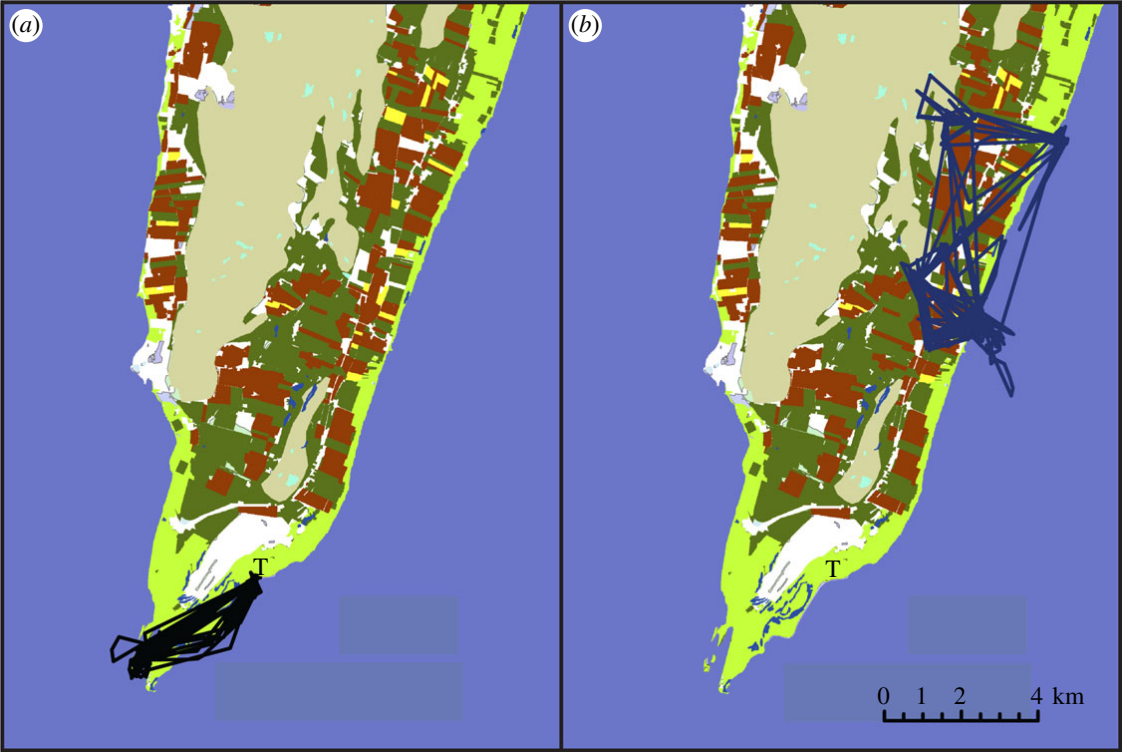
Examples of different types of movement behaviour in autumn-staging mallards for (*a*) ‘trap-dependent’ (i.e. returning to the trap) and (*b*) ‘trap-independent’ (i.e. not returning to the trap) individuals. T marks the location of the duck trap.

We also included three other explanatory covariates to control for extra sources of variation in movement metrics. First, the number of GPS locations is a function of the settings of the GPS units and the specific localities of the birds, and represents a technical bias influencing, for example, the minimum convex polygon (MCP) area, speed and travel distance of the birds. Therefore, the number of GPS locations (Reloc) was included as an explanatory covariate to account for varying sample size. For similar reasons, we excluded the last (incomplete) day/night of the total period for which each individual was followed. Second, a first inspection of the data revealed apparent differences in behaviour strategies among the mallards. They showed two main behaviours (Behtrap): ‘trap-dependent’ (i.e. returning to the trap on a daily basis; trapd) and ‘trap-independent’ (i.e. not returning to the trap; ntrap; figure 2). Third, mallard movements were expected to differ substantially between day (D) and night (N). Day/night was assigned based on solar angle of −6 degrees (which marks the end of civil twilight), i.e. when the sun was below this angle it was considered to be night and when above this angle it was considered day. As these two factors, trap behaviour and day/night movements (Behtrap and DN, respectively), are potentially important sources of variation in movement metrics, they were included by default as explanatory variables in the analyses.

The models were, therefore, as follows:

$$\text{log}(\text{Mov.metric})=\beta_{0}+\beta_{\mathrm{Inf}}+\beta_{T.\mathrm{aft}.\mathrm{Rel}}+\beta_{\mathrm{Reloc}}+\beta_{\mathrm{Behtrap}}+\beta_{\mathrm{DN}}+\beta_{\mathrm{Inf}*T.\mathrm{aft}.\mathrm{Rel}}+\varepsilon +\gamma ,$$

where

— *β*_0_ is the intercept, or reference, for the metrics recorded on uninfected trap-dependent mallards during the day. Trap-dependent observations are used as a reference as they were the most frequent in the data.
— *β*_Inf_ is the regression coefficient quantifying the effect of infection on the movement metrics compared with the reference *β*_0_, i.e. the metrics recorded on uninfected individuals.
— *β*_T.aft.Rel_ is the slope of the regression of the movement metrics against time after release in uninfected, trap-dependent mallards during the day.
— *β*_Reloc_ is the slope of the regression of the movement metrics against the number of relocations.
— *β*_Behtrap_ is the regression coefficient quantifying the difference between trap-independent mallards and the reference, i.e. trap-dependent individuals, on the movement metrics.
— *β*_DN_ is the regression coefficient quantifying the difference observed between night and the reference, i.e. day, on the movement metrics.
— *β*_Inf*T.aft.Rel_ is the regression coefficient quantifying the interaction between time after release and infection on the movement metrics. It is the difference in the slope of the regression of the movement metric against time after release between infected individuals and the reference, i.e. uninfected individuals.
— *ε* are the normal errors, with *ε*∼*N*(0,1).
— *γ* is the individual random term, accounting for variance heterogeneity among clustered observations recorded within a same individual.

## 3. Results

We did not find a difference in the ODBA values between LPAIV infected and uninfected mallards (overall mean values in table 2; *t*=0.538, d.f. = 19, two-sided paired *t*-test), nor did we find any statistical support for differences in the nine studied movement metrics between infected and uninfected individuals (electronic supplementary material, appendix S1). Among these, total distance travelled (*D*_tot_), average speed (*v*_seg_) and area covered by the animal (MCP) are three commonly studied and easily understood movement metrics. As we judged these to be the most biologically relevant, they are presented separately in table 3.

**Table 2.** Average overall dynamic body acceleration (ODBA) values (covering maximum 7 days) for infected versus uninfected mallards. A higher value stands for a higher amount of body movements. If a recaptured individual had changed infection status, it was counted as a new ODBA event. In total, 87 ODBA events were obtained from 37 mallards.

	ODBA	s.d.
infected	5559 (*n*=37)	1384
uninfected	5415 (*n*=50)	1338

**Table 3.** Average total cumulative distances (*D*_tot_ in m), mean speed between two consecutive locations (*v*_seg_ in m s^−1^), minimum convex polygon area (MCP in ha) and sample size (*n*, number of locations) during day/night and for each of the mallard categories during the first 7 days after sampling. trapd, trap-dependent individuals; ntrap, trap-independent individuals.

metric	trap	DN	uninfected	infected	*n*
*D*_tot_ (95% CI)	trapd	night	1462 (1255–1702)	1719 (2006–2473)	334
		day	2665 (2395–2965)	3134 (2800–3508)	320
	ntrap	night	2816 (2412–3286)	3311 (2818–3891)	136
		day	5133 (4620–5704)	6037 (5363–6796)	129
*v*_seg_ (95% CI)	trapd	night	0.04 (0.03–0.04)	0.04 (0.04–0.05)	334
		day	0.07 (0.06–0.08)	0.08 (0.07–0.09)	320
	ntrap	night	0.07 (0.06–0.08)	0.08 (0.07–0.10)	136
		day	0.13 (0.12–0.15)	0.15 (0.14–0.17)	129
MCP (95% CI)	trapd	night	1.19 (0.8–1.8)	1.9 (1.2–1.9)	334
		day	16.1 (12.0–21.5)	25.0 (18.4–34.0)	320
	ntrap	night	6.4 (4.3–9.7)	10.0 (6.5–15.5)	136
		day	87.1 (65.4–116)	135.7 (98.4–187.2)	129
*n*			669	250	

**Figure 3.**
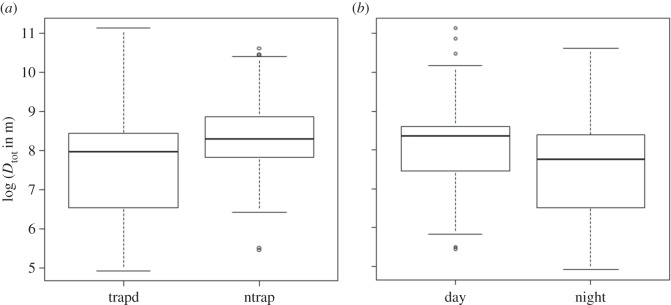
Total cumulative distances (*D*_tot_) in (*a*) trap-dependent (trapd) versus trap-independent (ntrap) mallards and (*b*) during day/night.

By contrast, and as expected, movement metrics differed greatly among the behavioural categories, as well as between day and night. The distance travelled by trap-independent mallards was higher than for trap-dependent individuals and movements were larger during day than night (figure 3). Uninfected trap-dependent mallards moved on average 2.7 km (95% CI: 2.4–3.0 km) each day, whereas movements for uninfected trap-independent individuals averaged 5.1 km (95% CI: 4.6–5.7 km; table 3). Comparing day and night revealed that MCP area for infected trap-independent mallards was 135.7 ha (95% CI: 98.4–187.2 ha) during day and 10.0 ha (95% CI: 6.5–15.5 ha) during night (table 3). With one exception, influence of day/night and behaviour (in relation to the trap) on mallard movement was always significant. The exception was the influence of day/night on the dispersion factor of Brownian movement (*h*) (electronic supplementary material, appendix S1).

For most metrics, models depicted a slight decrease of movements after release in uninfected individuals (see electronic supplementary material, appendix S1; e.g. slope for *D*_tot_ according to time after release for uninfected trap-dependent mallards during day: −0.03 ± 0.01, table 4). This negative relationship was also consistent in infected birds (e.g. slope difference in *D*_tot_ according to time after release for infected trap-dependent mallards relative to uninfected trap-dependent individuals: 0.01±0.02, table 4). As a result, the interaction between the infectious status and time after release was never significant for any of the movement metrics (electronic supplementary material, appendix S1). The absence of association between infection and movement was confirmed at the level of the intercept, i.e. just after release. At this time, and in contrast with our expectations, infected mallards tended to move more than uninfected individuals (e.g. intercept difference in *D*_tot_ for infected individuals relative to uninfected ones: 0.09±0.11, table 4) but that difference remained non-significant for all movement metrics (electronic supplementary material, appendix S1). The proportion of infected to uninfected mallards was 0.5 in both trap-dependent and trap-independent groups.

**Table 4.** Mean regression coefficients (*β*)±s.e. of the model log(Mov.metric) = Inf + T.aft.Rel + Reloc + Behtrap + DN + Inf*T. aft.Rel for total cumulative distance (*D*_tot_), mean speed between consecutive locations (*v*_seg_) and MCP in the first 7 days after sampling. Only coefficients describing the effect of infection are shown, i.e. *β*_0_ the intercept for uninfected mallards, *β*_inf_ the intercept difference between infected and uninfected individuals, *β*_T.aft.Rel_ the slope quantifying the movement metric evolution after release in uninfected mallards, and *β*_Inf*T.aft.Rel_ the slope difference between infected and uninfected individuals. *P*-values are indicated in brackets and significant values are in bold.

parameter	*D*_tot_	*v*_seg_	MCP
*β*_0_	**7.51**±**0.54** (***p***<**2**×**10**^−**16**^)	−**2.17**±**0.54** (***p***=**7**×**10**^−**5**^)	**9.82**±**1.45** (***p***=**2**×**10**^−**11**^)
*β*_inf_	0.09±0.11 (*p*=0.40)	0.11±0.11 (*p*=0.33)	0.15±0.29 (*p*=0.60)
*β*_T.aft.Rel_	−0.03±0.01 (*p*=0.06)	−0.02±0.01 (*p*=0.07)	−**0.11**±**0.04** (***p***=**3**×**10**^−3^)
*β*_inf*T.aft.Rel_	0.01±0.02 (*p*=0.58)	0.01±0.02 (*p*=0.64)	0.05±0.05 (*p*=0.38)

## 4. Discussion

We did not find any significant difference between LPAIV infected and uninfected mallards in how often, fast or far they moved during their migration stopover. Overall body movements, which can be viewed as agility, did also not differ between the two infection states. If the temporal effects of LPAIV infection are short-lived and/or slight, they can be hidden when measuring movements over a longer period of time, including times when birds are not infected. Therefore, we ran the analyses on 3 and 7 days of movement data, respectively, and concluded that results remained almost identical (see electronic supplementary material, appendix S2).

This suggests that LPAIV infection does not alter the behaviour of mallards. If true, this means that mallards are not impaired during stopover and can potentially migrate while carrying active LPAIV infection. This contradicts some previous studies, rejects our hypothesis, and, most importantly, has consequences for IAV epidemiology.

Previous studies on the effect of LPAIV infection on migration in waterfowl have been contradictory. There is some evidence that infection has an effect on swans. Wild Bewick's swans (*Cygnus columbianus bewickii*) naturally infected with LPAIV subtypes H6N2 and H6N8 made slower migratory progress, by leaving their wintering site more than a month after uninfected individuals. In addition to being delayed, they performed shorter migration flights and needed longer times for refuelling [44,45]. Infected swans also had lower reproductive success and lower return rate the following winter [45]. However, definite conclusions cannot be drawn from the small sample size (two infected versus four uninfected birds) in that study. In fact, when wild swans were experimentally infected no migratory differences could be detected compared with uninfected individuals [45].

In a Dutch study on wintering greater white-fronted geese (*Anser albifrons albifrons*), there was no difference in dispersal during the first 12 days after sampling between LPAIV infected and uninfected individuals [46]. Similarly, LPAIV infection status of mallards did not impact either recovery distance or stopover duration at Ottenby, Sweden [31], although juveniles trapped in September stayed longer while shedding increasing amounts of LPAIV.

Evidence for an impact of LPAIV infection on body mass in waterfowl is also equivocal. Latorre-Margalef *et al*. [31] reported on average 20 g lower body mass in infected mallards [31], whereas van Dijk *et al.* [47] showed that body mass did not differ between LPAIV infected and uninfected mallards when corrected for size, sex, age and migratory strategy [47]. Likewise, Kleijn *et al.* [46] found that infected and uninfected greater white-fronted geese did not differ in body mass in three out of four winters, but that infected birds had significantly lower body mass in the fourth [46]. Clearly, environmental conditions, seasonal effects and population differences may preclude unambiguous conclusions regarding the impact of LPAIV.

Several studies have proposed the possibility for long-distance spread of IAV by migrating waterfowl [18–22], but actual movement data on infected birds during migration or stopover are rare. Recently, van Dijk *et al.* [48] presented data where mallards with natural LPAIV infections performed less regional movements than uninfected individuals [48]. Sampling was performed twice (when deployments were attached and removed, respectively) and an association between infection and movement could only be detected during the last days of tracking, i.e. before removal of GPS loggers [48]. Infected and uninfected mallards did not differ with respect to daily local movements, which is in accordance with the data we have presented here.

Our study is unique in the sense that most mallards were sampled for infection several times, in some cases even on a daily basis, during the study period. Therefore, the same individual could contribute data as both infected and uninfected. In fact, this was often the case, which should decrease the risk for bias due to individual variation in movement behaviour. By obtaining more fine-grained data with respect to infection status than all previous studies on wild birds, our data more accurately reflect the natural cycling of mallards from infected to uninfected status over relatively short time periods during autumn stopover. We draw the conclusion that the infected mallards showed no impaired ability to spread LPAIV within the area used by mallards during stopover. That we show no effect of LPAIV infection on local/regional movements implies that the virus can be brought to the next destination along the migration route, which could either be another stopover locality or the final wintering area, where further spread to other individuals is possible. As shown previously (e.g. [19]), the short infection cycle of IAV makes it unlikely that a single duck spreads the virus along substantial parts of a migration route. Rather, spread over long distances must include transmission to other migrating individuals so that the virus travels in a ‘relay’ fashion. Our finding that mallards seem unaffected by LPAIV infection in terms of local/regional movements could indicate that LPAI viruses have co-evolved with this species.

The studied mallards may have experienced IAV infections before being sampled at Ottenby, and primary infections generally cause more profound effects than subsequent ones (e.g. [32]). However, even a primary infection that occurs before the onset of migration is likely to have small, if any, effect on movements during stopover. It should be noted that IAV infection alone may not have measurable effects on physical performance, but may have synergistic effects with other factors (e.g. poor body condition and infection with other pathogens). This could provide a partial explanation for the high inter-individual variation we observed with respect to movements. Alternatively, IAV infection could, at least partially, be the consequence of temporarily poor body conditions rather than the explanation for it [49]. Finally, while LPAIV infection may have little effect on movement, it could have other consequences not considered here. For example, infected ducks may be less wary, i.e. suffer a higher risk of predation, they may be less motivated to feed due to compromised health, or they may, on the contrary, feed more intensely or take more risks due to increased energy requirements to maintain physiological condition in the face of infection.

Clearly, further studies are needed to shed light on the effects of IAV infections in wild birds and to determine their role in global IAV dynamics. The approach we took here, using both GPS transmitters and accelerometers to gain an in-depth perspective of movement in ducks sampled intensively for IAV, has improved our knowledge of the impacts of LPAIV infection in mallards. In short, our results suggest that the mallard may be an ideal host for LPAI viruses, as movement behaviour remains unchanged during infection. This facilitates LPAIV spread among individuals at a certain site as well as to new areas along the flyway.

## Supplementary Material

ESM(1).doc that contains Table A1 which gives additional parameter estimates of the model over 7 days.

## Supplementary Material

ESM(2).doc that contains Table A2 which gives additional parameter estimates of the model over 3 days.

## Data accessibility

All GPS data are stored in Movebank (www.movebank.org) and accessible under the project name ‘Mallard Waldenstrom Ottenby Sweden’ (Movebank ID 3109235).
