# Supplementary material for: Does influenza A virus infection affect movement behaviour during stopover in its wild reservoir host?
Source: R Soc Open Sci. 2016 Feb 10;3(2):150633. doi: 10.1098/rsos.150633 (PMC4785985; doi:10.1098/rsos.150633)
Supplement: ESM(1).doc that contains Table A1 which gives additional parameter estimates of the model over 7 days. [file rsos150633supp1.docx]

Table A1. Regression coefficient estimates (*βs*) ± SE of the model Log(*Mov.metric*) = *Inf + T.aft.Rel + Reloc + Behtrap + DN + Inf*T.aft.Rel* for nine movement metrics investigated for seven days after release. Student t- and p-values are indicated in brackets; ddf = 886. Significant parameters are indicated in bold.

| ***Parameter*** | ***D_tot_*** | ***D_max_*** | ***d_seg_*** | ***v_seg_*** | ***v_max_*** | ***FPT_coef_*** | ***FPT_int_*** | ***h*** | ***MCP*** |
| --- | --- | --- | --- | --- | --- | --- | --- | --- | --- |
| ***β_0_*** | **7.51±0.54**  **(13.78, *< 2e-16*)** | **6.37±0.98**  **(6.51, *1e-10*)** | **4.71±0.54**  **(8.66, *<2e-16*)** | **-2.17±0.54**  **(-4.0, *7e-05*)** | -0.49±0.95  (-0.52, *0.60*) | **1.49±0.18**  **(8.24, *6e-16*)** | **1.74±0.60**  **(2.92, *4e-03*)** | **14.29±4.75**  **(3.01, *3e-03*)** | **9.82±1.45**  **(6.80, *2e-11*)** |
| ***β_inf_*** | 0.09±0.11  (0.86, *0.40*) | 0.13±0.20  (0.20, *0.51*) | 0.09±0.11  (0.86, *0.39*) | 0.11±0.11  (0.97, *0.33*) | 0.20±0.19  (1.04, *0.30*) | 0.02±0.04  (0.43, *0.67*) | 0.02±0.12  (0.24, *0.81*) | 1.36±0.85  (1.44, *0.15*) | 0.15±0.29  (0.53, *0.60*) |
| ***β_T.aft.Rel_*** | -0.03±0.01  (-1.87, *0.06*) | -0.05±0.02  (-2.0, *0.05*) | -0.03±0.01  (1.84, *0.07*) | -0.02±0.01  (-1.79, *0.07*) | -0.02±0.02  (-1.02, *0.31*) | 4e-03±4e-03  (0.98, *0,33*) | 0.00±0.01  (0.05, *0.96*) | -0.09±0.11  (-0.82, *0.41*) | **-0.11±0.04**  **(-3.01, *3e-03*)** |
| ***β_Reloc_*** | 0.01±0.01  (0.90, *0.37*) | 0.02±0.02  (0.78, *0.44*) | -0.01±0.01  (0.75, *0.46*) | -0.01±0.01  (-0.62, *0.54*) | 0.01±0.02  (0.62, *0.53*) | **-0.02±4e-03**  **(-47.0, *4e-06*)** | **0.11±0.01**  **(7.62, *6e-14*)** | -0.15±0.12  (-1.33, *0.18*) | 0.06±0.04  (1.78, *0.08*) |
| ***β_Behtrap_*** | **0.66±0.09**  **(7.02, *4e-12*)** | **0.71±0.17**  **(4.53, *2e-05)*** | **0.66±0.09**  **(7.04, *4e-12*)** | **0.66±0.09**  **(7.10, *3e-12*)** | **0.61±0.16**  **(3.76, *2e-04*)** | **-0.31±0.03**  **(-10.28,  *<2e-16*)** | **0.75±0.10**  **(7.48, *2e-13*)** | **2.87±0.78**  **(3.66, *3e-04*)** | **1.69±0.25**  **(6.74, *3e-11*)** |
| ***β_DN_*** | **-0.60±0.19**  **(-3.14, *2e-03*)** | **-1.11±0.34**  **(-3.23, *1e-03*)** | **-0.60±0.19**  **(3.14, *2e-03*)** | **-0.63±0.19**  **(-3.29, *1e-03*)** | **-0.98±0.33**  **(-2.96, *3e-03*)** | **0.57±0.06**  **(8.94, *< 2e-16*)** | **-1.58±0.21**  **(-7.55, *1e-13*)** | -0.29±1.67  (-0.17, *0.86*) | **-2.60±0.51**  **(-5.14, *3e-07*)** |
| ***β_inf*T.aft.Rel_*** | 0.01±0.02  (0.55, *0.58*) | 0.03±0.04  (0.83, *0.41*) | 0.01±0.02  (0.55, *0.58*) | 0.01±0.02  (0.47, *0.64*) | 0.01±0.04  (0.14, *0.89*) | -8e-03±-7e-03  (-1.29, *0.20*) | 1e-03±0.02  (0.05, *0.96*) | 0.01±0.17  (0.06, *0.95*) | 0.05±0.05  (0.89, *0.38*) |
