# Supplementary material for: Does influenza A virus infection affect movement behaviour during stopover in its wild reservoir host?
Source: R Soc Open Sci. 2016 Feb 10;3(2):150633. doi: 10.1098/rsos.150633 (PMC4785985; doi:10.1098/rsos.150633)
Supplement: ESM(2).doc that contains Table A2 which gives additional parameter estimates of the model over 3 days. [file rsos150633supp2.docx]

Table A2. Regression coefficient estimates (*βs*) ± SE of the model Log(*Mov.metric*) = *Inf + T.aft.Rel + Reloc + Behtrap + DN + Inf*T.aft.Rel* for nine movement metrics investigated for three days after release. Student t- and p-values are indicated in brackets; ddf = 745. Significant parameters are indicated in bold.

| ***Parameter*** | ***D_tot_*** | ***D_max_*** | ***d_seg_*** | ***v_seg_*** | ***v_max_*** | ***FPT_coef_*** | ***FPT_int_*** | ***h*** | ***MCP*** |
| --- | --- | --- | --- | --- | --- | --- | --- | --- | --- |
| ***β_0_*** | **7.60±0.58**  **(13.09, *< 2e-16*)** | **6.60±1.08**  **(6.14, *1e-09*)** | **4.81±0.58**  **(8.30, *<5e-16*)** | **-2.08±0.58**  **(-3.6, *4e-04*)** | -0.31±1.04  (-0.30, *0.77*) | **1.35±0.19**  **(7.22, *1e-12*)** | **2.22±0.60**  **(3.70, *2e-04*)** | **12.23±4.34**  **(2.82, *5e-03*)** | **10.37±1.55**  **(6.69, *5e-11*)** |
| ***β_inf_*** | 0.21±0.16  (1.32, *0.19*) | 0.43±0.29  (1.47, *0.14*) | 0.21±0.16  (1.32, *0.19*) | 0.24±0.16  (1.53, *0.13*) | 0.54±0.28  (1.92, *0.06*) | 0.07±0.05  (1.35, *0.18*) | -0.13±0.16  (-0.78, *0.44*) | **2.95±1.18**  **(2.50, *0.01*)** | 0.60±0.42  (1.41, *0.16*) |
| ***β_T.aft.Rel_*** | -0.03±0.03  (-1.06, *0.29*) | -0.05±0.06  (-0.92, *0.36*) | -0.03±0.03  (-1.07, *0.29*) | -0.03±0.03  (-0.99, *0.32*) | -0.01±0.06  (-0.18, *0.86*) | **0.02±0.01**  **(2.09, *0.04*)** | -0.04±0.03  (-1.16, *0.25*) | -0.29±0.24  (-1.23, *0.22*) | -0.14±0.08  (-1.68, *0.09*) |
| ***β_Reloc_*** | 0.01±0.01  (0.76, *0.45*) | 0.01±0.03  (0.56, *0.58*) | -0.01±0.01  (-0.78, *0.44*) | -0.01±0.01  (-0.66, *0.51*) | 0.01±0.03  (0.43, *0.67*) | **-0.02±5e-03**  **(-4.09, *5e-05*)** | **0.10±0.01**  **(7.15, *2e-12*)** | -0.10±0.11  (-0.92, *0.36*) | 0.05±0.04  (1.42, *0.16*) |
| ***β_Behtrap_*** | **0.74±0.10**  **(7.31, *7e-13*)** | **0.83±0.19**  **(4.44, *1e-05)*** | **0.74±0.10**  **(7.34, *6e-13*)** | **0.74±0.10**  **(7.39, *4e-13*)** | **0.73±0.18**  **(4.03, *6e-05*)** | **-0.36±0.03**  **(-11.56,  *<2e-16*)** | **0.89±0.10**  **(8.73, *<2e-16*)** | **3.06±0.74**  **(4.15, *4e-05*)** | **1.95±0.27**  **(7.22, *1e-12*)** |
| ***β_DN_*** | **-0.69±0.20**  **(-3.43, *6e-04*)** | **-1.26±0.38**  **(-3.36, *8e-04*)** | **-0.69±0.20**  **(-3.42, *7e-04*)** | **-0.72±0.20**  **(-3.56, *4e-04*)** | **-1.15±0.36**  **(-3.18, *2e-03*)** | **0.61±0.07**  **(9.33, *< 2e-16*)** | **-1.72±0.21**  **(-8.20, *1e-15*)** | -0.95±1.52  (-0.63, *0.53*) | **-2.87±0.54**  **(-5.30, *2e-07*)** |
| ***β_inf*T.aft.Rel_*** | -0.04±0.05  (-0.65, *0.52*) | -0.09±0.10  (-0.94, *0.35*) | -0.04±0.05  (-0.65, *0.52*) | -0.05±0.05  (-0.84, *0.40*) | -0.14±0.10  (-1.45, *0.15*) | -0.03±-0.02  (-1.83, *0.07*) | 0.06±0.06  (1.00, *0.32*) | -0.62±0.40  (-1.54, *0.12*) | -0.14±0.14  (-0.94, *0.35*) |
